# Non-parametric synergy modeling of chemical compounds with Gaussian processes

**DOI:** 10.1186/s12859-021-04508-7

**Published:** 2022-01-06

**Authors:** Yuliya Shapovalova, Tom Heskes, Tjeerd Dijkstra

**Affiliations:** 1 Radboud University, Postbus 9010, 6500 GL Nijmegen, The Netherlands; 2 Max Planck Institute for Developmental Biology, Max-Planck-Ring 25, 72076 Tübingen, Germany; 3 Department for Women’s Health, University Clinic Tübingen, Calwerstrasse 7, 72076 Tübingen, Germany; 4 Translational Bioinformatics, University Clinic Tübingen, Schafhausenstrasse 77, 72072 Tübingen, Germany

**Keywords:** Combination therapy, Synergy, Gaussian processes, Hand model

## Abstract

**Background:**

Understanding the synergetic and antagonistic effects of combinations of drugs and toxins is vital for many applications, including treatment of multifactorial diseases and ecotoxicological monitoring. Synergy is usually assessed by comparing the response of drug combinations to a predicted non-interactive response from reference (null) models. Possible choices of null models are Loewe additivity, Bliss independence and the recently rediscovered Hand model. A different approach is taken by the MuSyC model, which directly fits a generalization of the Hill model to the data. All of these models, however, fit the dose–response relationship with a parametric model.

**Results:**

We propose the Hand-GP model, a non-parametric model based on the combination of the Hand model with Gaussian processes. We introduce a new logarithmic squared exponential kernel for the Gaussian process which captures the logarithmic dependence of response on dose. From the monotherapeutic response and the Hand principle, we construct a null reference response and synergy is assessed from the difference between this null reference and the Gaussian process fitted response. Statistical significance of the difference is assessed from the confidence intervals of the Gaussian process fits. We evaluate performance of our model on a simulated data set from Greco, two simulated data sets of our own design and two benchmark data sets from Chou and Talalay. We compare the Hand-GP model to standard synergy models and show that our model performs better on these data sets. We also compare our model to the MuSyC model as an example of a recent method on these five data sets and on two-drug combination screens: Mott et al. anti-malarial screen and O’Neil et al. anti-cancer screen. We identify cases in which the HandGP model is preferred and cases in which the MuSyC model is preferred.

**Conclusion:**

The Hand-GP model is a flexible model to capture synergy. Its non-parametric and probabilistic nature allows it to model a wide variety of response patterns.

**Supplementary Information:**

The online version contains supplementary material available at 10.1186/s12859-021-04508-7.

## Background

Assessing synergy and antagonism of chemical compounds has applications in medicine, pharmacology and ecotoxicology. The advantage of combining synergetic drugs is that they can reach higher effects while having lower side effects or toxicity in comparison to a single drug. Similarly, antagonistic drugs reach smaller effects compared to the prediction from their individual potencies. Understanding of synergy allowed the development of combination therapies [1] which proved useful in various areas, including treatment of cancer [2] and asthma [3]. In ecotoxicology, this led to an understanding of how toxins interact and, in particular, how they can affect a human body [1].

By comparing the expected non-interactive (null) and observed responses, one can assess whether there is synergy or antagonism between two drugs. The most common candidates for the non-interactive response models are the Loewe additivity [4] and Bliss independence [5] models. However, there are other candidates such as the Highest Single Agent (HSA) [6], the Tallarida [7] and the recently rediscovered Hand model [8]. Sinzger et al. [8] present detailed theoretical comparisons of the popular null models including comparisons between the isoboles of the corresponding null models.

Loewe additivity and Bliss independence models often serve as bases for various extensions that incorporate more complex interaction patterns. Jonker et al. [9] developed models for testing level-dependent and ratio-dependent synergy/antagonism. Level-dependent synergy/antagonism occurs when the difference (between non-interactive response and observed response) at low doses deviates from the difference at high doses. For example, antagonism can be observed at low doses at synergy and high doses. Ratio-dependent synergy/antagonism happens when, say, antagonism is observed when the mixture is dominated by drug 1 and synergy when the mixture is dominated by drug 2. Wicha et al. [10] study asymmetric interactions of drugs. In particular, they define perpetrator and victim drugs. Perpetrators cause a change of the half-maximal effective concentration, $EC_{50}$, of the other drug in the mixture, and victims are affected by this change. Both Jonker et al. [9] and Wicha et al. [10] develop methods for both Loewe additivity and Bliss independence type models.

Most null reference models—in particular Loewe additivity, Tallarida, and Hand models—are based on monotherapeutic dose–response curves. Frequently, the Hill curve is chosen for modeling the monotherapeutic dose–response relationship [11]. Some studies have shown that other choices of monotherapeutic dose–response curves might be preferable in some cases [12, 13], but the Hill curve is the most common. Importantly, all these models are parametric, meaning that they specify a fixed set of possible shapes as defined by the range of the parameters. Parametric models have advantages in that they are generally more interpretable than non-parametric models and perform well when the data follow the pattern implied by the parametric model [12, 13]. However, parametric models also have well-documented disadvantages, the most important one being their fixed set of possible shapes when data behave differently from the parametric model assumptions. Hence, non-parametric models and especially Gaussian process (GP) models have become popular recently. For example, even in cases where a good but high-dimensional model is available from physics or engineering, GPs have found applications as the workhorse of surrogate modeling [14]. Thus, the GP framework appears to be a natural approach, especially for complicated systems like a biological cell’s response to a perturbation. We combine the flexible GP approach to dose–response surface modeling with the Hand principle to construct the null reference model. The Hand model was shown to satisfy biochemically desirable principles [8]. Thus, combining the GP framework with the Hand principle results in a flexible data-driven model—Hand-GP—which satisfies desired biological assumptions. In recent work, Ronneberg et al. [15] use Gaussian processes in combination with the Bliss model. However, although the Bliss model is convenient in terms of simplicity of the computations, it does not satisfy some desirable principles of null models, including the sham combination principle, as discussed in Sinzger et al. [8].

The Bliss and the HSA are two standard non-parametric models that do not require fitting parametric monotherapeutic curves to estimate the reference surface. Both of these models require only knowledge of the effect for single doses to estimate the predicted effect. However, the Bliss and the HSA models do not satisfy the sham combination property nor the associative property. The sham combination property states that a drug can be neither synergistic nor antagonistic when combined with itself. The associative property implies that combining combinations of drugs is equivalent to combining the drugs directly [8]. Additionally, since the dose–response curve is not fitted in these models, it is hard to estimate either the measurement noise or the uncertainty of the predicted effect. This is a key difference with the non-parametric Hand-GP model as it naturally allows to compute the uncertainty of the model parameters and the predicted effect.

As main competitor to our Hand-GP model, we use the recent MuSyC model [16, 17] as this (1) also fits the entire response surface and (2) is highly parametric, with 12 parameters to specify the full model. The advantage of this model is that the parameters are interpretable and can be related to the hypothetical underlying mass-action rate equations [16]. Of the 12 parameters, 5 relate to synergy. Thus, a second advantage is that complicated synergy patterns can be captured in the parameters, say antagonism in efficacy (the effect for high doses) and synergy in potency (the 50% effect dose, $EC_{50}$). For comparison, our Hand-GP model has only 4 hyperparameters, one of which captures the noise level, i.e. the lack of fit. We follow common machine learning terminology where the parameters of the GP are called hyperparameters [18] because they can be interpreted as such in a Bayesian setting. In particular, the kernel hyperparameters can change the prior distribution over functions.

Our proposed model is based on Gaussian processes and is non-parametric. A Gaussian process is completely defined by its mean and kernel functions. Different kernels can be used to express different structures observed in the data [18]. We propose a new kernel optimized to capture the logarithmic dependence of the effect on compound dose in biochemical systems. As an extra benefit, the length scale hyperparameters of this kernel allow for data-adapted plotting of response curves and surfaces, striking a middle ground between linear and logarithmic axes. In contrast to standard approaches to synergy [4–7], we fit a GP surface to the complete dose–response matrix instead of fitting only the monotherapeutic data. This helps with the estimation of the observational noise and with the uncertainty quantification. Also, the estimated monotherapeutic response curves are more robust. We construct the null reference model numerically using the Hand model [8] by locally inverting the GP-fitted monotherapeutic data. Synergy is then assessed by a synergy effect surface as the difference between the GP-fitted response surface and the Hand-constructed null reference surface. This synergy effect surface allows for different effects at different dose combinations, for example, dose–dependent synergy.

## Results

We compare the performance of the Hand-GP model with the MuSyC model. We also provide the results of the original analysis with the Loewe, Bliss, and Median Effect models when relevant. We analyse the performance of the models on three simulated and two experimental data sets. In detail, we use a simulated data set from Greco et al. [19] to which we refer as the Greco data, two data sets from our own hand (one with strong synergy and one with strong antagonism), and two experimental data sets used by Chou et al. [20] to showcase their Median Effect model to which we refer as the Chou and Talalay data.

All the data sets are inhibitory, meaning a larger compound dose leads to a smaller response. In the Hand-GP model, we quantify synergy by taking the difference between the response surface (GP fitted to the raw response data) and the null reference model, generated by the Hand construction from the monotherapeutic GP fitted response data. As generally speaking synergy is the desired effect, we subtract the GP-fitted response data from the null reference. Then, a positive difference means a smaller response than expected from the null reference, or equivalently a larger effect, i.e. more inhibition.

We fit the MuSyC model using the Python library synergy [21]. In the MuSyc model the response is fitted with a 12-parameter model. To make sure that the parameters of MuSyC model are reasonably estimated, in particular that the estimated range of the parameter $E_{max}$ is within limits [0,100] or [0,1] depending on the application, we limited the parameters $E_{1}, E_{2}, E_{3}$ to be in [0, 100] or [0, 1]. Synergy is determined from a subset of the parameters, termed $\beta$, $\alpha _{12}$, $\alpha _{21}$, $\gamma _{12}$ and $\gamma _{21}$. Parameter $\beta$ corresponds to a change in synergistic efficacy, i.e. at large doses of both drugs the effect is $\beta$ larger. Parameters $\alpha$ correspond to a change in the effective dose and $\gamma$ to a change in the Hill slope coefficient. To enable a better comparison of the Hand-GP model with the MuSyC model, we also fit a constrained MySyc model with $\alpha _{12} = \alpha _{21} = \gamma _{12} = \gamma _{21} = 1$ and $\beta = 0$. This constrained MuSyC model serves as an equivalent null reference model for direct comparison to our Hand-GP model. Subtracting the 12-parameter MuSyC model from the constrained MuSyC model, we obtain an effect surface for comparison to the effect surface of the Hand-GP model.

**Fig. 1:**
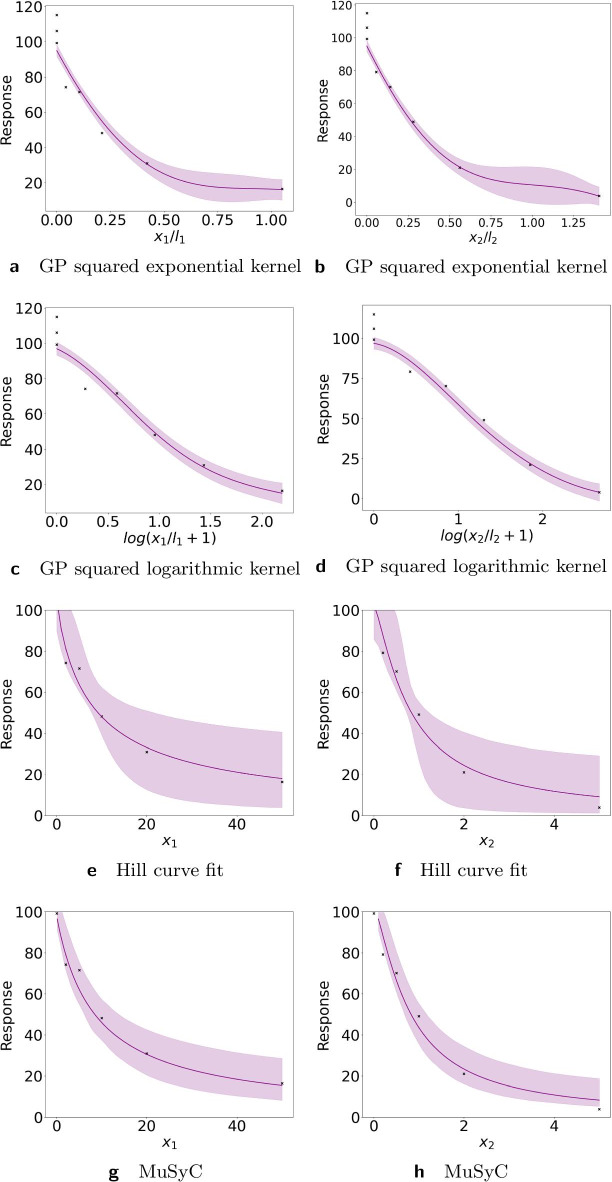
Dose–response curves for the Greco data using the squared exponential kernel (top row,** a**,** b**), the logarithmic squared exponential kernel (second from top,** c**,** d**), the Hill curve (third from top,** e**,** f**) and the MuSyC model (bottom row,** g**,** h**). For both GPs with different kernels and for the MuSyC model we fit the complete dose–response surface, and plot monotherapeutic slices. Left side: results for drug 1, right side: results for drug 2. Results from both GP regressions are plotted on their natural scale: as (linear) $x_i/l_i$ for the squared exponential kernel and as $\log (x_i/l_i+1)$ for the logarithmic squared exponential kernel. Note the difference in the estimates of the uncertainty

One of our contributions is the logarithmic squared exponential kernel, tailored to dose–response modeling. In Fig. 1 we compare fits with our new kernel to fits with the standard (linear) squared exponential kernel. We also provide the fits with Hill curve and MuSyC model. The data in this figure come from Greco et al. [19] and are discussed in more detail in the next section. The mean squared errors (MSE’s) of the fits with the logarithmic squared exponential kernel (72.01 for drug 1 and 63.35 for drug 2) are considerably lower than those of the fits with the (linear) squared exponential kernel (91.52 for drug 1 and 72.1 for drug 2). This shows that a Gaussian process with the logarithmic squared exponential kernel can approximate these data better. We provide a similar comparison for both simulated data (Greco, LA synergy, LA antagonism) and real data (Chou and Talalay data) in Additional file 1: Table S1. Results in Additional file 1: Table S1 show that the logarithmic kernel performs better than the linear one for 3 of the 5 data sets considered. In particular it performs better in cases where the data were generated on the logarithmic scale. Figure 1e, f also illustrate fits of the Hill curve. The MSEs are lower than with the logarithmic squared exponential kernel with values of 16.55 and 13.37. This is expected since the data were generated from the Hill equation. We used the parametric bootstrap [22] to obtain confidence intervals for the fitted Hill curves. The resulting confidence intervals are considerably larger than those obtained with the Gaussian processes approach. The large confidence intervals in the fitted Hill curve come from the large confidence intervals of the parameters of the Hill curves. In particular for drug A and Fig. 1e the estimates of the parameters and corresponding 95% confidence intervals are $E_0=105.14$ [90.4; 110], $E_{max}=0.0$ [0.0; 29.5], $h=0.87$ [0.65; 2.57], $C=8.16$ [3.25; 13.71]. Similarly for drug B the confidence intervals for the parameters are large $E_0=102.79$ [87.4; 110], $E_{max}=0.0$ [0.0; 26.15], $h=1.27$ [0.86; 5.65], $C=0.79$ [0.47; 1.21] which results in large confidence intervals for the fitted Hill curve of drug B in Fig. 1f. Figure 1g, h illustrate monotherapeutic slices of the fits obtained with the MuSyC model. The MSE is the highest for the MuSyC model for drug A, 110.4, while for drug B the MSE, 62.7, is comparable to the GP fit and is even marginally better.

### Greco simulated data

In this section, we illustrate the performance of the model on the benchmark data from Table 3 of Greco et al. [19]. The data set was generated with mild Loewe synergism (synergy coefficient 0.5), which in many regions of the response surface corresponds to mild Bliss antagonism. The data have a $6\times 6$ design. Greco et al. [19] considered 13 different models and reported detailed results for Loewe additivity and Bliss independence models which we compare to the Hand-GP and MuSyC models in Table 1. The Hand-GP model (third column of Table 1) predicts synergy except for a single dose combination $(x_1=5, x_2=5)$ where it predicts antagonism. The MuSyC model (fourth column of Table 1) predicts antagonism for 15 dose combinations and synergy for 10. The Hand-GP model appears to capture synergism even better than the Loewe model (fifth column of Table 1) although the data were simulated from this model, as the Loewe model incorrectly predicts antagonism for four dose combinations. The poorer performance of the Loewe model can be explained by measurement noise which was added to the data. Since the effect was constructed to be only mildly synergistic, measurement noise can affect the predictions for some dose combinations. Additionally, as shown in Sinzger et al. [8], the Hand isoboles are very close to those of the Loewe model, but non-increasing dose–response curves can lead to mild Loewe antagonism and to Hand synergism. The last (sixth) column of Table 1 shows results from Bliss independence which predicts antagonism except for three dose combinations. As the data were simulated from a Loewe model these results are not surprising and are discussed in detail by Greco et al. [19]. We present parameter estimates and overall effect estimates for both Hand-GP and MuSyC model in Table 2. We created the overall effect measure from the difference of the volumes under the surfaces of the regular GP model and the null reference Hand-GP model. The volume under the surface is approximated using Delaunay triangulation [23]. This effect measure is presented in the lower block of Table 2. In this table, as well as in later tables, we color-code synergism as green, antagonism as red and additivity (no interaction effect) as grey. The MuSyC model has five parameters that correspond to different types of synergism/antagonism, thus each of them is color-coded separately. A MuSyC model that corresponds to no interaction, so just an additive effect, has parameters $\beta = 0$ and $\alpha _{12} = \alpha _{21} = \gamma _{12} = \gamma _{21} = 1$. We can see from Table 2 that additivity is predicted by all parameters of the MuSyC model as the additive effect values are within the 95% confidence intervals of each of these five parameters. Interestingly, the volume difference indicates synergy for the MuSyC model, while each of the parameters indicates additivity. The uncertainty about the surface in the case of the Hand-GP model leads to additivity in terms of the volume difference. From Table 1 we see that the predictions from the MuSyC model are somewhat in between those of the Loewe and Bliss models. As it is discussed in Greco et al. [19] in some areas a small degree of Loewe synergism corresponds to a small degree of Bliss antagonism, so general disagreement between the models is not surprising.

**Table 1:**
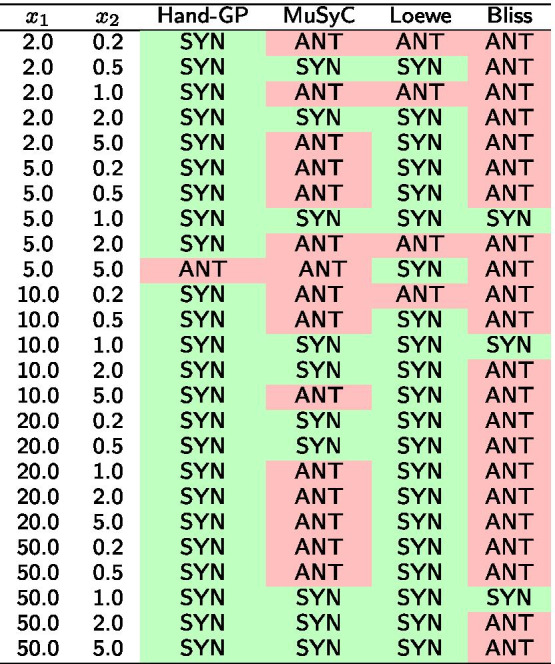
Comparison of Hand-GP model to the MuSyC, Loewe and Bliss models from Greco [19] on the Greco simulated data

Green denotes synergy and red denotes antagonism

**Table 2:**
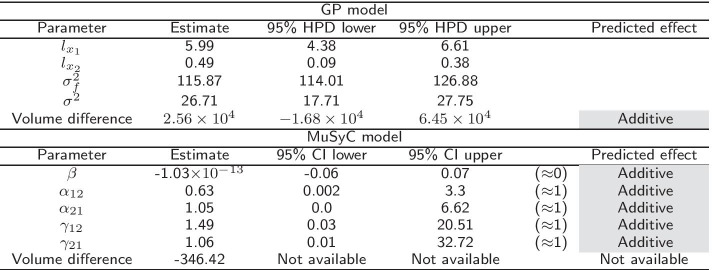
Parameters of the GP and the MuSyC models for the Greco data

Also reported are highest posterior density (HPD) estimates from Bayesian inference of the GP and confidence intervals (CI) from maximum likelihood estimation of the MuSyC model. Green denotes synergy, red denotes antagonism and grey denotes additivity

**Fig. 2:**
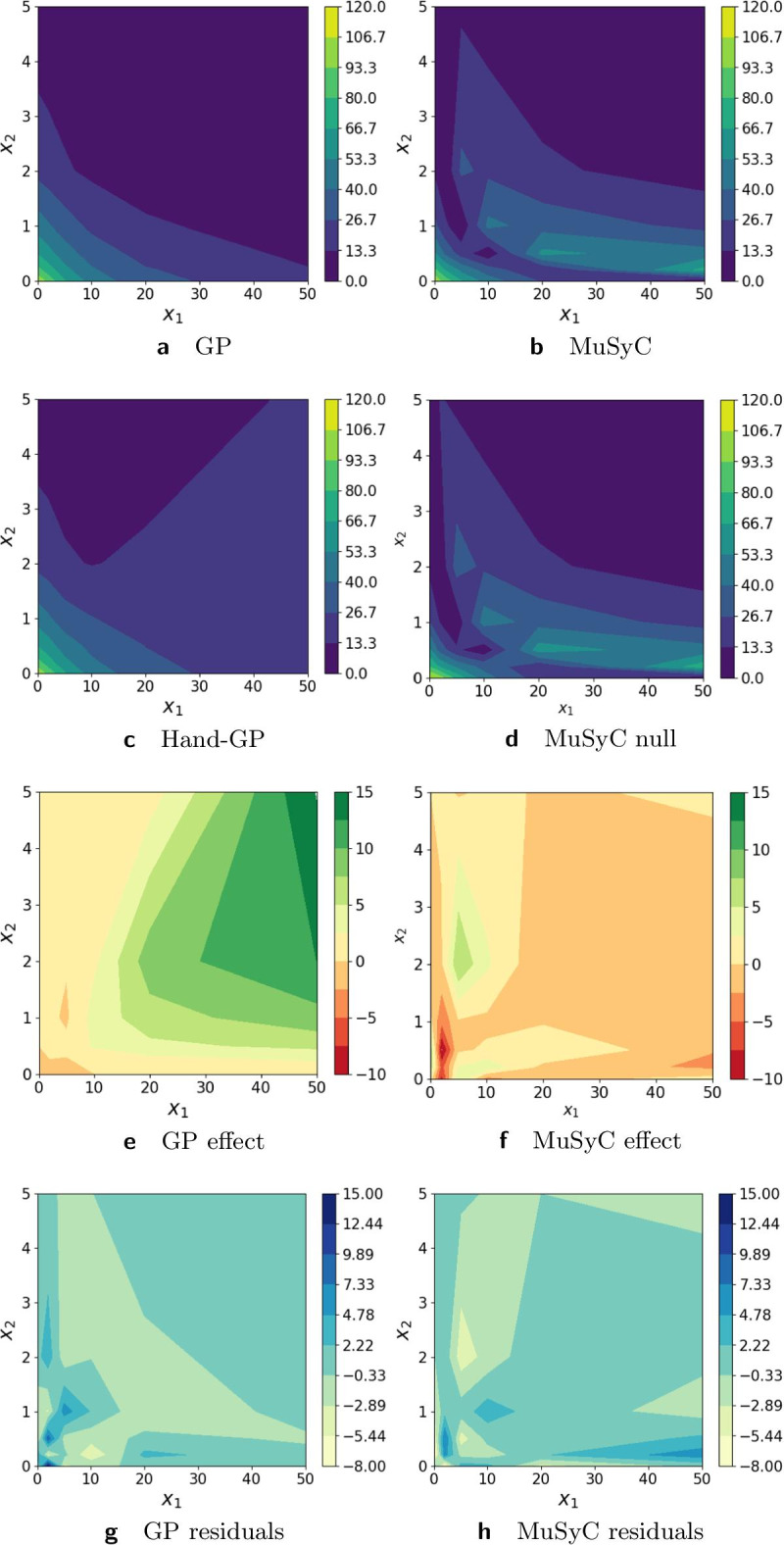
Analysis of Greco simulated data with the Hand-GP (left column,** a**,** c**,** e**,** g**) and MuSyC (right column,** b**,** d**,** f**,** h**) models. Top row (**a**,** b**) shows the fitted response surfaces. For Hand-GP this is a fit to the non-parametric GP model; for MuSyC a fit to the parametric MuSyC model. The second row (**c**,** d**) shows the null reference models. For Hand-GP this is the Hand construction derived from the fitted monotherapeutic responses from the top row; for MuSyC a fit to a constrained MuSyC model. The third row (**e**,** f**) shows the synergistic effect surfaces as the difference between the first and the second row. The bottom row (**g**,** h**) shows the residuals, the difference between the data and the fits from the top row

In Fig. 2, we provide a more detailed analysis of the differences between the Hand-GP and MuSyC models. Note that in Fig. 2 we present the results on the linear scale for both models to make them directly comparable. In the following figures, we plot the Hand-GP model on the logarithmic scale since it is the natural presentation for the newly proposed kernel. The third row shows the synergistic effect surfaces as the difference between the first and second row: green (positive) indicates a synergistic effect and red (negative) indicates an antagonistic effect. We see that the Hand-GP model predicts synergy almost everywhere, whereas the MuSyC model predicts both synergy and antagonism. The bottom row shows the residuals, the difference between the data and the models in the top row. The mean squared errors for the whole surface are similar for the GP and MuSyC models, 13.07 and 13.71 respectively. As can be seen from Fig. 2g, the residuals are higher for the GP model around zero doses, but the overall landscape of the residuals appears marginally better for the GP model.

### Robustness of the results to the experimental design

**Fig. 3:**
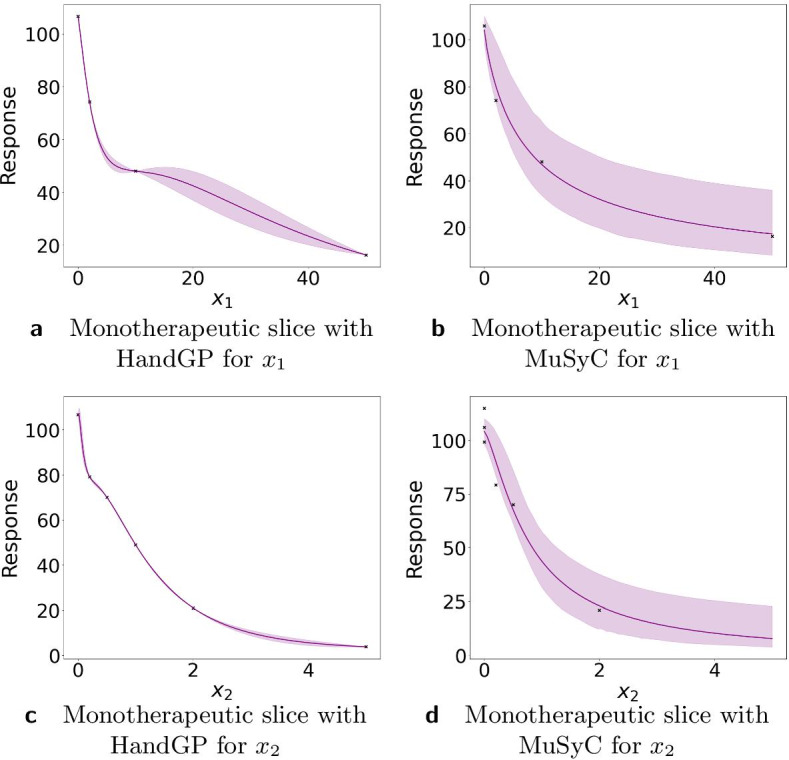
Analysis of the Greco simulated data with reduced $4 \times 6$ design with the Hand-GP (left column,** a**,** c**) and the MuSyC (right column,** b**,** d**) models. For the Hand-GP model we fit the whole surface and plot monotherapeutic slices. The confidence intervals for the MuSyC model are obtained with the parametric bootstrap

**Fig. 4:**
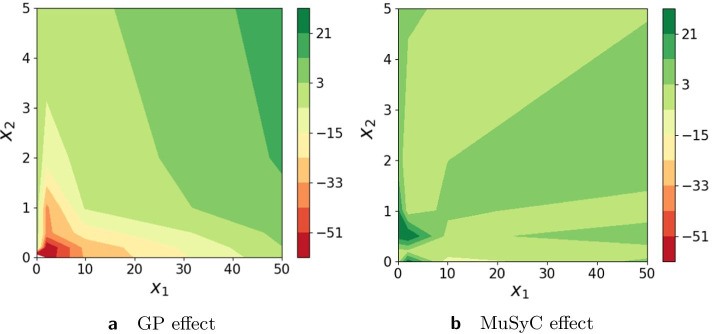
Analysis of Greco simulated data with a reduced $4 \times 6$ design with the Hand-GP (left,** a**) and MuSyC (right,** b**) models. Both figures show the effect surfaces as the difference between the fitted response surface and the null model

In this section we analyze the robustness of the synergy estimates to the experimental design of the Greco data set, in particular to reduction of the number of doses. The original matrix design is $6 \times 6$ with six doses for drug A [0, 2, 5, 10, 20, 50] and six for drug B [0, 0.2, 0.5, 1, 2, 5]. We reduced the data to $4 \times 6$ with four doses for drug A [0, 2, 10, 50] and six doses for drug B [0, 0.2, 0.5, 1, 2, 5]. Figure 3 illustrates monotherapeutic slices of the surfaces for the MuSyC model (Fig. 3a, b) and for Hand-GP model (Fig. 3c, d). We obtained confidence intervals for the MuSyC model with the parametric bootstrap [22] and for the Hand-GP model from Eq. (6). One can see that the confidence intervals are reasonably narrow in comparison to those obtained with Hill curves as presented in Fig. 1e, f. Uncertainty for drug $x_1$ in case of the Hand-GP model increases as can be seen from Fig. 3c. Figure 4 illustrates the effect surfaces of both models. We observe that the effect of the Hand-GP model becomes more extreme: mildly synergistic areas become more synergistic and mildly antagonistic areas become more antagonistic. For the MuSyC model, the effect switches from mostly mild antagonism to stronger synergy. This example illustrates the importance of the design matrix. With the reduced matrix design of $4 \times 6$ we get stronger effects in both models in comparison to the $6\times 6$ design. Generally, nonparametric approaches require more data, and for the Hand-GP model we recommend using at least $6\times 6$ or $8\times 8$ designs. Smaller design matrices are also not ideal for the MuSyC model as the number of data points approaches the number of parameters in this case.

### Simulated data with Loewe synergy and antagonism

**Fig. 5:**
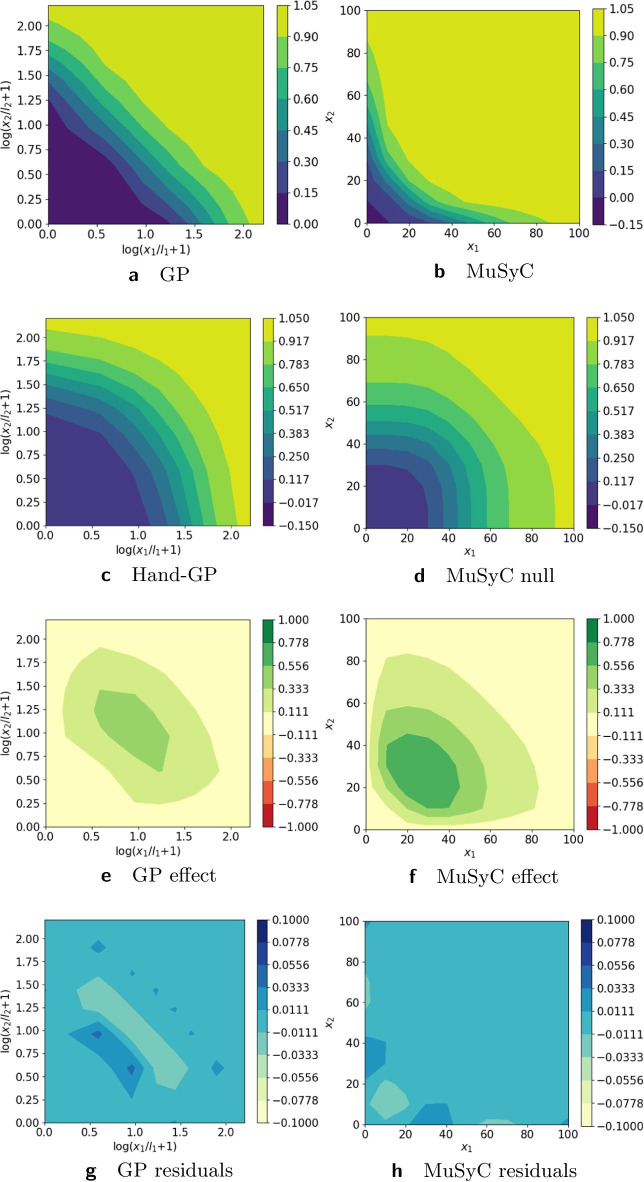
Analysis of Loewe synergy simulated data with Hand-GP (left column,** a**,** c**,** e**,** g**) and MuSyC (right column,** b**,** d**,** f**,** h**) models. Top row (**a**,** b**) shows the fitted response surfaces. For Hand-GP this is a fit to the non-parametric GP model; for MuSyC a fit to the parametric MuSyC model. The second row (**c**,** d**) shows the null reference models. For Hand-GP this is the Hand construction derived from the fitted monotherapeutic responses from the top row; for MuSyC a fit to a constrained MuSyC model. The third row (**e**,** f**) shows the synergistic effect surfaces as the difference between the first and second row. The bottom row (**g**,** h**) shows the residuals, the difference between the data and the fits from the top row

The Greco data set was only mildly synergistic, so we generated two data sets with stronger effects (synergistic and antagonistic). To highlight the differences between the models we used an $11\times 11$ design without noise. Details of the parameter values used to simulate the data can be found in the Additional file 1. Figure 5 and Table 3 present results for the synergistic data set. Both the Hand-GP and the MuSyC models predict only synergy since the effect surface is always positive, unlike Fig. 2 where both the Hand-GP and the MuSyC model predicted antagonism for some doses. The stronger synergy is reflected in the volume difference of the Hand-GP model which was $2.57\times 10^{4}$ for the Greco data and now is $2.06\times 10^{5}$. The volume difference of the MuSyC model indicates stronger synergy than the Hand-GP model with the measure being $4.0\times 10^{5}$. Curiously, while the effect surfaces in Fig. 2 show stronger estimated synergy for the MuSyC model which is confirmed by the volume difference measure, the synergy parameters in Table 3 indicate antagonism in both the efficacy parameter $\beta$ and the Hill slope parameters $\gamma _{12}$ and $\gamma _{21}$.

**Table 3:**
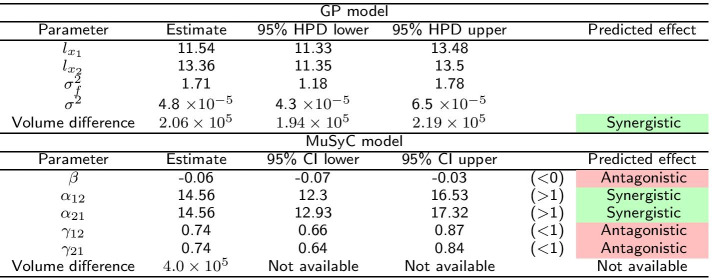
Parameters of the GP and MuSyC models for Loewe synergy

Also reported are highest posterior density (HPD) estimates from Bayesian inference of the GP and confidence intervals (CI) from maximum likelihood estimation of the MuSyC model

**Fig. 6:**
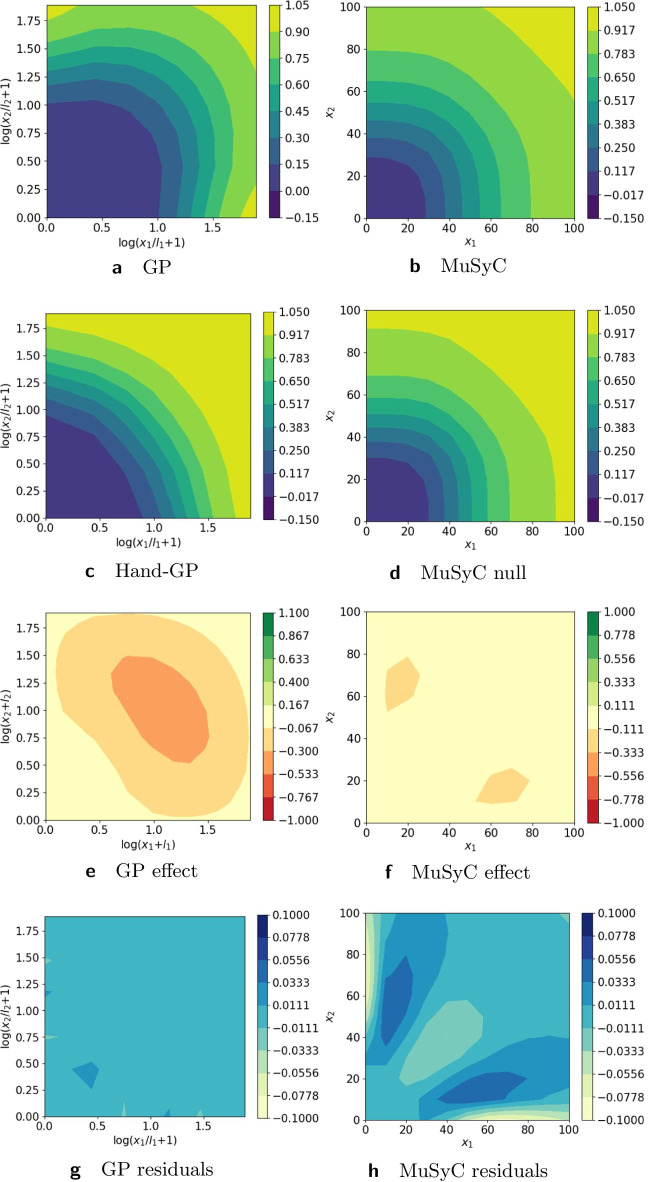
Analysis of Loewe antagonism simulated data set with Hand-GP (left column,** a**,** c**,** e**,** g**) and MuSyC (right column,** b**,** d**,** f**,** h**) models. Top row (**a**,** b**) shows the fitted response surfaces, for Hand-GP this is a fit to the non-parametric GP model; for MuSyC a fit to the parametric MuSyC model. The second row (**c**,** d**) shows the null reference models. For Hand-GP this is the Hand construction derived from the fitted monotherapeutic responses from the top row; for MuSyC a fit to a constrained MuSyC model. The third row (**e**,** f**) shows the synergistic effect surfaces as the difference between the first and second row. The bottom row (**g**,** h**) shows the residuals, the difference between the data and the fits from the top row

Figure 6 and Table 4 present results for the antagonistic data set. The Hand-GP model generally predicts antagonism which is also confirmed in the summary measure of volume difference in Table 4. All parameters of the MuSyC model indicate additivity. The volume difference measure, however, indicates antagonism.

**Table 4:**
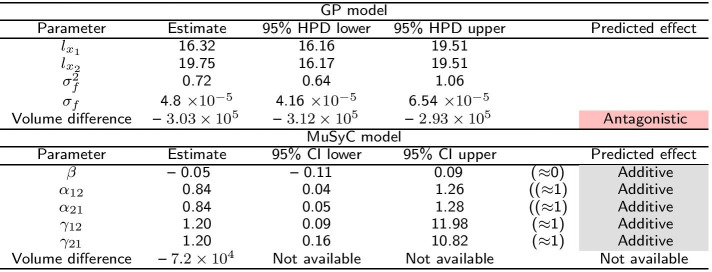
Parameters of GP and MuSyC models for Loewe antagonism

Also reported are highest posterior density (HPD) estimates from Bayesian inference of the GP and confidence intervals (CI) from maximum likelihood estimation of the MuSyC model

### Experimental data from Chou and Talalay

The data were initially published by Yonetani and Theorell in 1964 [24] and re-analyzed in Chou and Talalay in 1984 [20]. The data are from a $6\times 6$ design and are examples of mutually exclusive and non-exclusive inhibitors. In our analysis, we compare the Hand-GP model to the MuSyC model and the reproduced combination index from the Median Effect model of Chou and Talalay 1984 [20, Figure 3, 5]. We follow the analysis of the Median Effect model as indicated in [25, Tab. 1, 2]. In detail, we show predictions from all models for the diagonal rays only and plot them as a function of the (fitted) fractional effect. However, while the Median Effect model is only fitted to the monotherapeutic and diagonal ray data in three separate fits, the Hand-GP and MuSyC models are fit once to all 36 dose–response data points.

### Inhibition of alcohol dehydrogenase by two mutually exclusive inhibitors

**Fig. 7:**
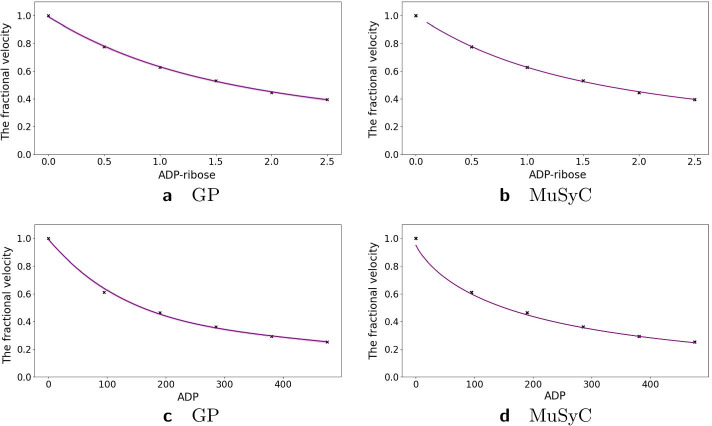
Analysis of mutually exclusive inhibitors data set with with Hand-GP (left column,** a**,** c**) and MuSyC (right column,** b**,** d**) models. The figure illustrates monotherapeutic slices from the estimated surfaces. Response is the fractional inhibition of horse liver alcohol dehydrogenase. Note that confidence intervals are indicated in all panels but are so small as to be almost invisible

**Fig. 8:**
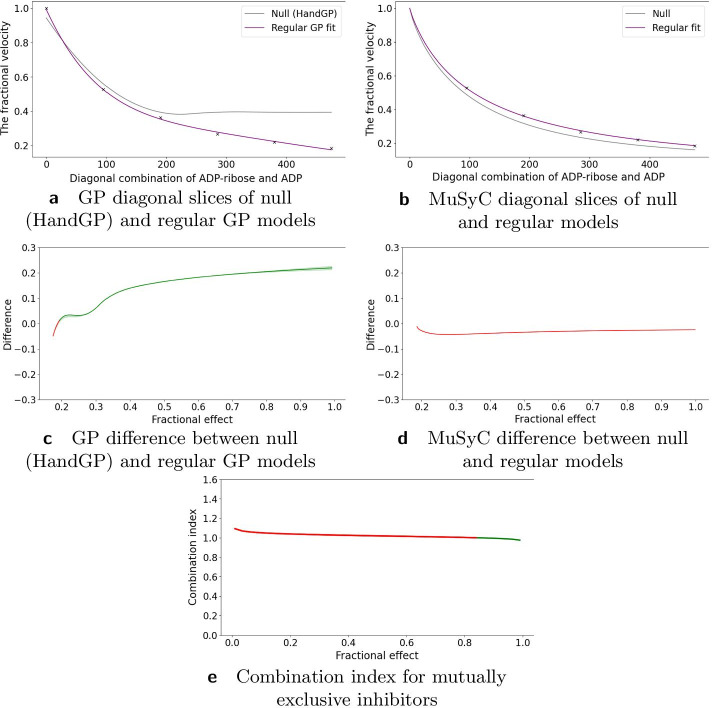
Analysis of mutually exclusive inhibitors data with Hand-GP (left column,** a**,** c**) and MuSyC (right column,** b**,** d**) models. Estimated effect with the original Median Effect model analysis is shown in the bottom plot,** e**. Top row (**a**,** b**) shows fitted slices of the surfaces along the diagonal (in grey) and the null reference models along the same diagonal (in purple). The middle row (**c**,** d**) shows the difference between the regular and null models, plotted as a function of fitted fractional effect (inverse of the purple curve in top panels). Estimated synergy is shown in green and antagonism in red. Note that confidence intervals are indicated for the HandGP model but are so small as to be almost invisible. Bottom panel (**e**) shows the combination index reproduced from [20]

The first study in Yonetani et al. [24] concerned the inhibition of horse liver alcohol dehydrogenase by two mutually exclusive inhibitors: ADP-ribose and ADP and was re-analyzed in Chou et al. [20] using the Median Effect model. In Fig. 7 we see that both the MuSyC and the Hand-GP models fit monotherapeutic data well. In Fig. 8a, c we can see that the Hand-GP model predicts antagonism at low doses of both drugs (along the diagonal ray) as the GP fitted curve lies below the null reference. Conversely, the Hand-GP model predicts synergy at high doses of both drugs as the GP fitted curve lies above the null reference. In Fig. 8e we present the combination index reproduced from Chou et al. [20] which shows the switch from antagonism to synergy as well, although the combination index is close to 1 which indicates additivity. The MuSyC model predicts mostly antagonistic effects which are close to additivity with a large uncertainty in the parameter $\beta$ and an unidentified upper bound for the parameter $\gamma _{12}$ which predicts synergy. Such uncertainty can be due to the large number of parameters (12) of the MuSyC model relative to the number of data points (36). Alternatively, a more conceptual explanation can be provided in this case (we thank an anonymous reviewer for this suggestion). In this example we consider a mutually exclusive pair of drugs as also reflected in the estimates of the MuSyC model: $\alpha _{12} \approx \alpha _{21} \approx 0$. This means that when one drug is active, the dose of the other drug is multiplied by $\sim 0$. Thus, the other drug in this case is blocked. This principle is well illustrated in Figure 2 of Wooten et al. [26]: when $\alpha = 0$, $\beta$ (which is based on E3) and gamma drop out of the MuSyC equation, thus they may take any value without impacting the fit quality. So when it happens that one parameter’s value causes another parameter to not matter, the latter parameter will always have a wide confidence interval. The results from the Hand-GP model qualitatively agree with the results from Yonetani et al. [24]: at lower doses indicating antagonism and at higher doses indicating synergy. All of the models indicate that the overall effect is close to zero (or combination index close to 1 for the Median Effect model) which indicates additivity of the inhibitory effects of ADP-ribose and ADP. The volume difference summary measure indicates mild synergism for the Hand-GP model and antagonism for MuSyc model. Note the different scales of the Hand-GP and the MuSyC model in comparison to the scale of the combination index reproduced from Chou et al. [20] (Tables 5, 6).

**Table 5:**
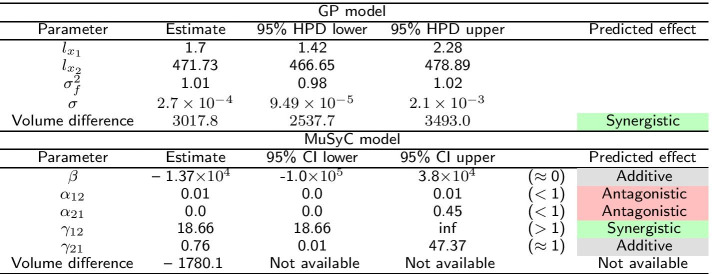
Parameters of GP and MuSyC models for mutually exclusive inhibitors

Also reported are highest posterior density (HPD) estimates from Bayesian inference of the GP and confidence intervals (CI) from maximum likelihood estimation of the MuSyC model

### Inhibition of alcohol dehydrogenase by two mutually non-exclusive inhibitors

**Fig. 9:**
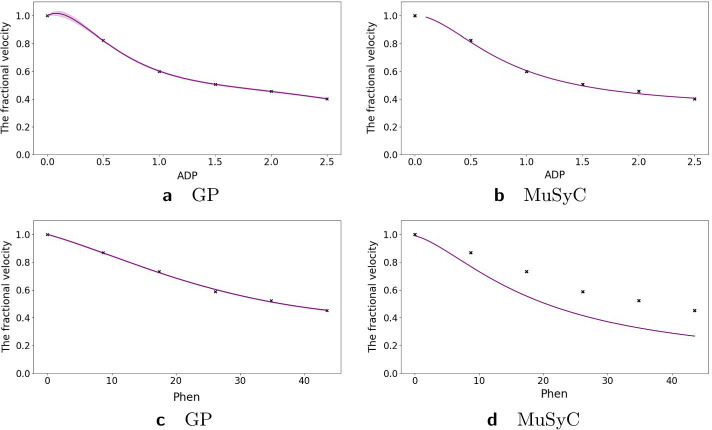
Analysis of mutually non-exclusive inhibitors data set with with Hand-GP (left column,** a**,** c**) and MuSyC (right column,** b**,** d**) models. The figure illustrates monotherapeutic slices from the estimated surfaces. The response is the fractional inhibition of horse liver alcohol dehydrogenase, $x_1$ is the concentration *o*-phenanthroline and $x_2$ the concentration ADP

**Fig. 10:**
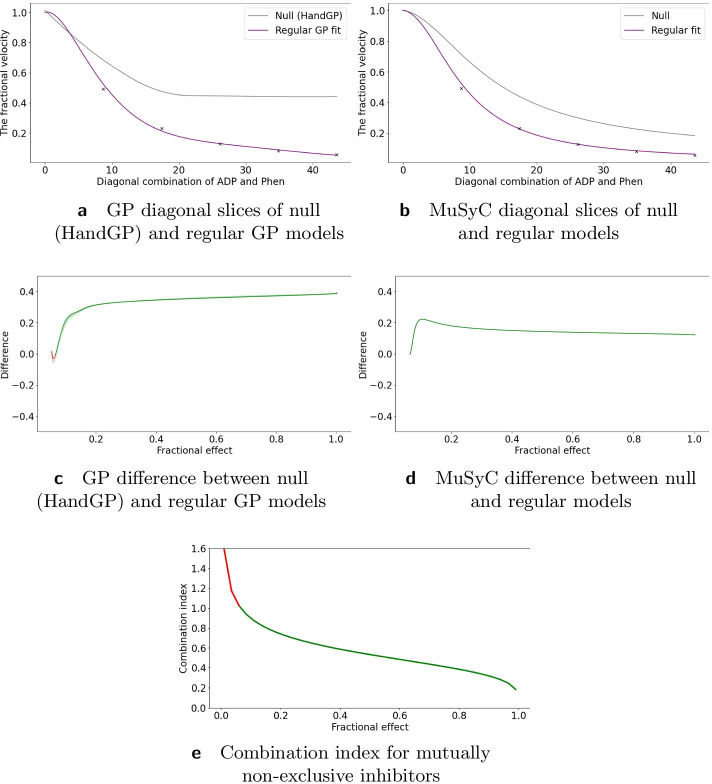
Analysis of mutually non-exclusive inhibitors data with Hand-GP (left column,** a**,** c**) and MuSyC (right column,** b**,** d**) models. Estimated effect with the original Median Effect model analysis are shown at the bottom plot. Top row shows the fitted slices of the surfaces along the diagonal. For Hand-GP this is a fit to the non-parametric GP model; for MuSyC a fit to the parametric MuSyC model. Corresponding null models are in grey and regular models in purple. The middle row shows the difference between predicted effects by regular and null models. Estimated synergy effect is shown in green and antagonism in red. Note that confidence intervals are indicated for the HandGP model but are so small as to be almost invisible. Bottom panel,** e**, shows the combination index reproduced from [20]

The second study in Yonetani et al. [24] concerned the inhibition of horse liver alcohol dehydrogenase by two competitive, mutually non-exclusive inhibitors: *o*-phenanthroline and ADP and was re-analyzed in Chou et al. [20] using the Median Effect model. In Fig. 10e we present the combination index reproduced from Chou et al. [20]. Using the Hand-GP model we obtain antagonism for the combination of low doses and synergism for the combination of high doses which agrees with the analysis in Chou et al. [20]. The MuSyC model predicts synergy for the parameters $\alpha _{12}$ and $\alpha _{21}$ (change of effective dose) and antagonism for $\gamma _{12}$ and $\gamma _{21}$, change of the Hill coefficient. Figure 10d illustrates that along the diagonal MuSyC model predicts only synergy. The volume difference measure is comparable for both models and indicates mild synergy. Complete surface analysis for both models can be found in the Additional file 1. In Fig. 9 we can see that the MuSyC model does not fit the monotherapeutic part of the data well, while the GP fits look good. Both models fit the diagonal of the surface quite well, which is illustrated in Fig. 10. In this case the results of the Hand-GP model agree with the analysis in Chou et al. [20].

**Table 6:**
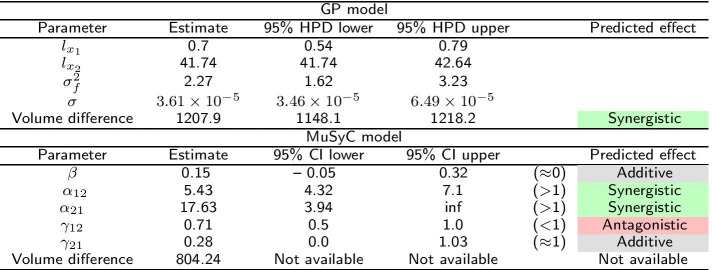
Parameters of GP and MuSyC models for mutually non-exclusive inhibitors

Also reported are highest posterior density (HPD) estimates from Bayesian inference of the GP and confidence intervals (CI) from maximum likelihood estimation of the MuSyC model

## Assessing the models on drug combination screens

In previous sections, we have compared the proposed Hand-GP model and the MuSyC model on multiple simulated and real-world data examples, which are commonly used to assess synergy models. In this section, we consider two drug combination screens. The first data set we consider is the Mott et al. anti-malarial screen [27]. The second data set is the O’Neil et al. anti-cancer screen [28] for which we analyze one cell line (isolate HB30). Both data sets were considered in the recent MuSyC paper by Wooten et al. [17]. We look at the volume measure to find examples that are similar and dissimilar between the two models. Figure 17 illustrates scatter plots of the volume measure for both data sets and both models. Note that we do not expect widespread agreement between the two models according to the volume measure. As shown in the five detailed examples from the previous sections, the volume measure disagreed in two out of five cases. Additionally, the dose–response matrix design differs between the two data sets. The Mott et al. anti-malarial screen has $6\times 6$ and $10 \times 10$ design matrices, while the O’Neil et al. anti-cancer screen has a $4\times 4$ design matrix. We can see from Fig. 17a, b that the smaller design matrix results in a noisier estimate of the volume measure. This agrees with our earlier conclusion from the Greco data set with reduced matrix design: we reduced the experimental design from $6\times 6$ matrix to $4 \times 6$ matrix and observed that both models produced biased estimates.

**Fig. 11:**
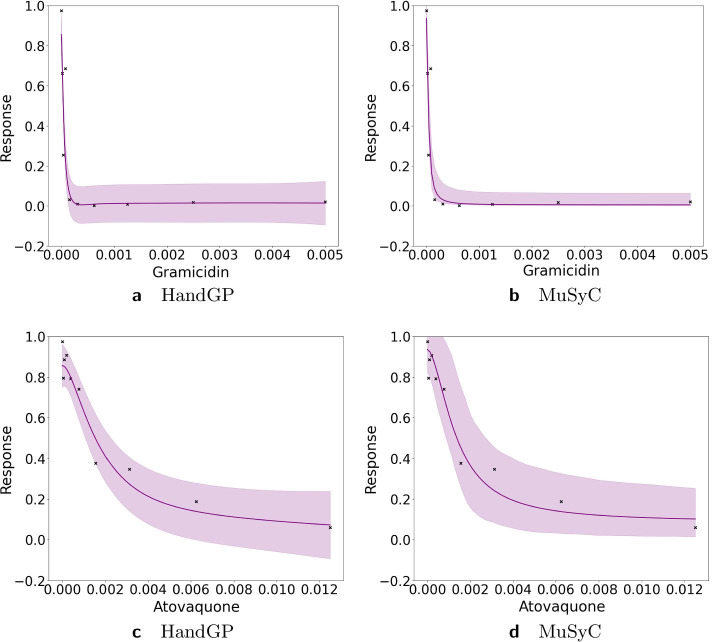
Monotherapeutic slices for the combination of Gramicidin and Atovaquone for isolate HB30 (Mott et al. anti-malarial screen). Left column (**a**,** c**) shows monotherapeutic slices of the Hand-GP model and right column (**b**,** d**) shows monotherapeutic slices of the MuSyC model

**Fig. 12:**
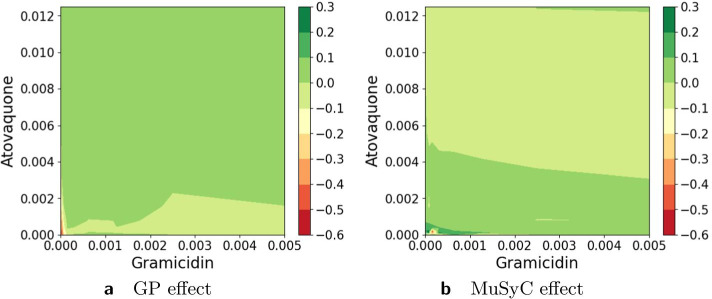
Predicted effect for the combination of Gramicidin and Atovaquone for islate HB30 (Mott et al. anti-malarial screen). On the left,** a**, prediction by the Hand-GP model, on the right,** b**, prediction by the MuSyC model. This example demonstrates general agreement between the Hand-GP and MuSyC models

**Fig. 13:**
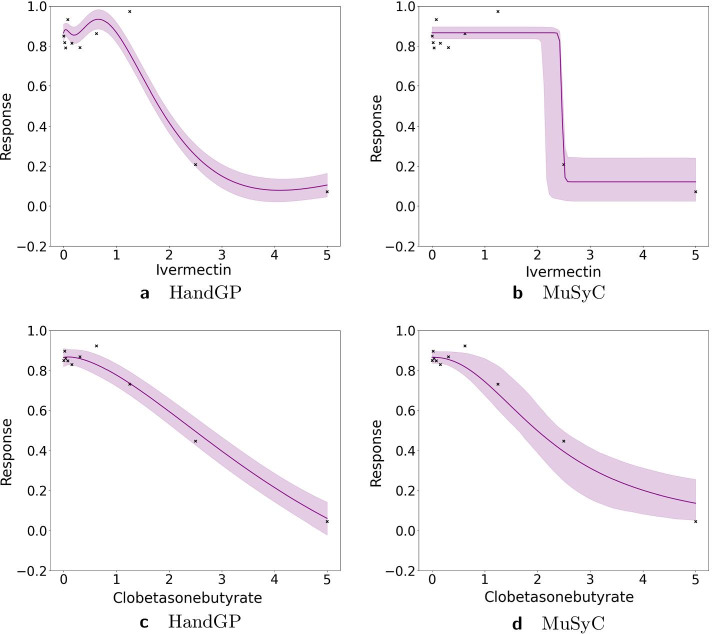
Monotherapeutic slices for the combination of Ivermectin and Clobetasonebutyrate for isolate HB30 (Mott et al. anti-malarial screen). Left column (**a**,** c**) shows monotherapeutic slices of the Hand-GP model and right column (**b**,** d**) shows monotherapeutic slices of the MuSyC model

**Fig. 14:**
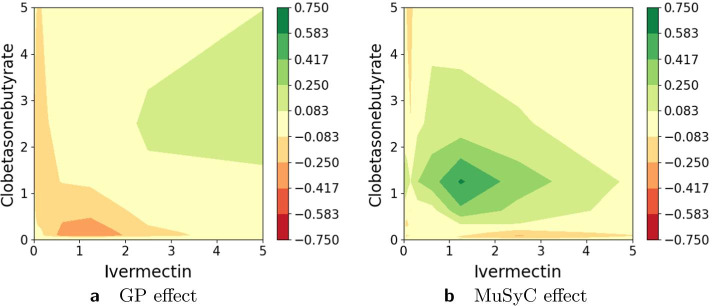
Predicted effect for the combination of Ivermectin and Clobetasonebutyrate for isolate HB30 (Mott et al. anti-malarial screen). On the left,** a**, prediction by the Hand-GP model, on the right,** b**, prediction by the MuSyC model. This example demonstrates disagreement between HandGP and MuSyC models

**Fig. 15:**
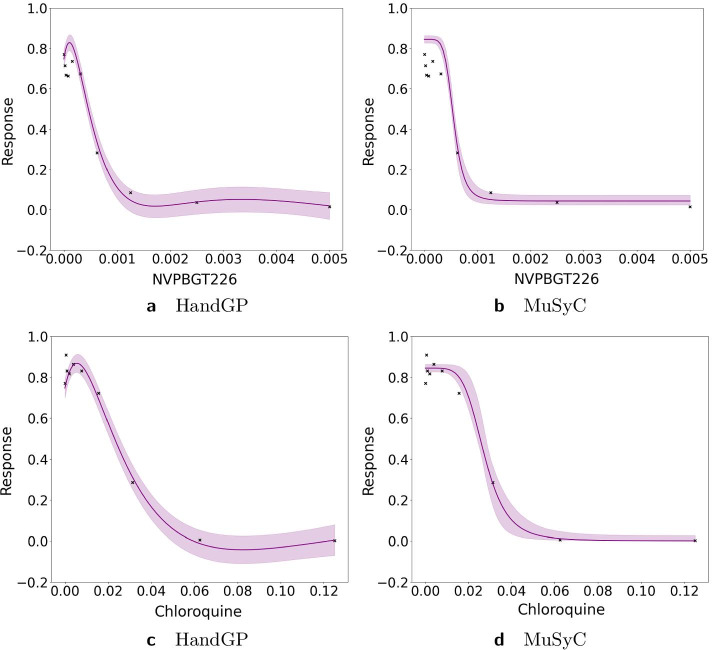
Monotherapeutic slices for the combination of NVPBGT226 and Chloroquine for isolate 3D70 (Mott et al. anti-malarial screen). Left column (**a**,** c**) shows monotherapeutic slices of the Hand-GP model and right column (**b**,** d**) shows monotherapeutic slices of the MuSyC model

**Fig. 16:**
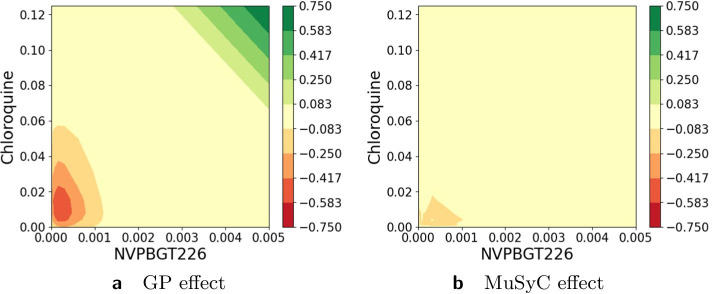
Predicted effect for the combination of NVPBGT226 and Chloroquine for the cell line 3D70 (Mott et al. anti-malarial screen). On the left,** a**, prediction by Hand-GP model, on the right,** b**, prediction by MuSyC model. This example demonstrates disagreement between Hand-GP and MuSyC models in some cases

**Fig. 17:**
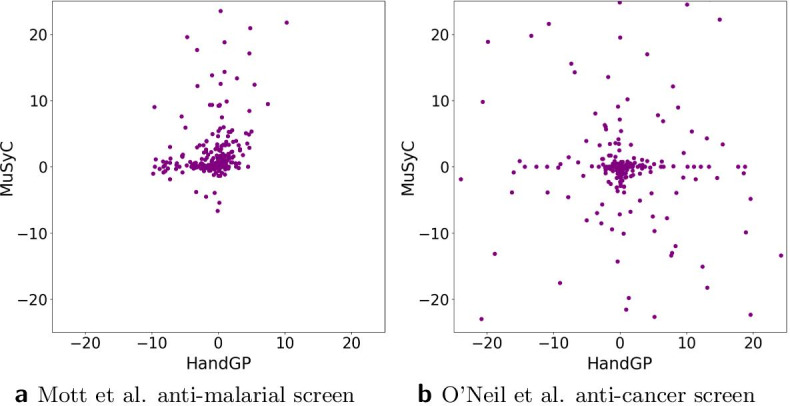
Scatter plots for the volume measure obtained with the Hand-GP (x-axis) and the MuSyC (y-axis) models. On the left,** a**, for the Mott et al. anti-malaria screen, on the right,** b**, for the O’Neil et al. anti-cancer screen

Figures 11, 12, 13, 14, 15, 16 and 17 present three illustrative examples for the Mott et al. anti-malarial screen data: an example where both models agree, and two examples with disagreement each favoring a different model. The first example, for the combination of Gramicidin and Atovaquone for isolate HB30, is illustrated in Figs. 11 and  12. We see from Fig. 12 that both models predict overall synergy and minor antagonism at very low doses. Generally, the HandGP and the MuSyC models agree about the predicted effect in this example. Figure 11 shows that the estimated monotherapeutic curves are very similar for the example of Gramicidin and Atovaquone combination. The second example, the combination of Ivermectin and Clobetasonebutyrate for isolate HB30, is illustrated in Figs. 13 and 14. From Fig. 14 we see that the HandGP predicts minor antagonism at lower doses, the near-zero effect at mid-range doses, and mild synergy at higher doses. The MuSyC model predicts synergy at mid-range doses. Thus, generally, models disagree. In Fig. 13 we observe that one of the monotherapeutic curves for the MuSyC models has a non-smooth drop in the effect which is likely to lead to this disagreement. The final example is the combination of NVPBGT226 and Chloroquine for isolate HB30, the results for this combination are presented in Figs. 15 and  16. As we can see from Fig. 15 the GP exhibits non-monotonic behavior at very low and at very high doses. This translates into different predicted effects in Fig. 16: synergy at high doses and antagonism at low doses. We provide similar examples for O’Neil et al. anti-cancer screen in Additional file 1: S4–S9.

## Discussion

We introduced the Hand-GP model, a combination of the Hand principle for constructing null reference models with a Gaussian process using a new logarithmic squared exponential kernel. We demonstrated the performance of the Hand-GP model on multiple benchmark data sets, both simulated and experimental ones, and two drug combination screens. We compared the Hand-GP model with the recent parametric MuSyC model and with the well-established Loewe, Bliss, and Median Effect models. In all cases of the benchmark data sets, the Hand-GP model performed very well, and we obtained expected predictions of synergistic effects. It is important to note that different models define synergy differently, thus the claim of predicting the "correct" effect can be misleading. Nevertheless, since the Hand model is biochemically plausible [8] and Gaussian processes are a flexible non-parametric framework for curve fitting and uncertainty quantification, we think the Hand-GP model can be advantageous in many real-world applications. In two drug combination screens, Mott et al. anti-malarial screen [27] and O’Neil et al. anti-cancer screen [28], we identified cases where the HandGP model is preferred and where the MuSyC is preferred. Our comparison of the proposed method to the MuSyC model shows that parametric models - although useful and interpretable-cannot account for some dose–response surfaces that are observed in practice. If the experimental data deviate too much from the assumed parametric curve/surface, then the predicted effect can be incorrect. In such situations, a non-parametric approach can be of help to predict an interaction effect. In this paper we restrict our attention to smooth kernels optimized for data on a logarithmic scale. However, our approach can be extended with, for example, the linear-Matern kernel [29] which can be beneficial for non-smooth or non-stationary data.

While we showcased the Hand-GP model for binary and time-fixed compound interactions, the model can be easily extended to include more than two compounds or time as an additional component in the Gaussian process. Especially time has received only limited attention in the synergy literature, but see [30]. More generally, viewing time as part of the phenotype, there can be many different types of changes in phenotype in response to perturbations, e.g. changes of cell shape or spatial rearrangements of the cell. All these changes can be flexibly modeled within the GP framework, leveraging research in the machine learning community [31, 32]. Further extensions are possible to incorporate various experimental scenarios. For example, when the data are noisy and thus the observations are non-monotonic, as in O’Neil et al. anti-cancer screen [28], a constrained Gaussian processes framework can be used [33–35] such that monotonicity is enforced in the GP. Ultimately, we view our model proposal as a building block in a pipeline that predicts the phenotype of a cell type in response to multiple perturbations. The flexibility of Gaussian processes makes such a vision more plausible than parametric modeling approaches.

## Conclusion

In this paper, we proposed a non-parametric approach to dose–response synergy modeling based on Gaussian processes and the Hand principle. We proposed a new kernel function that operates on the log-scale of the doses and takes into account the specifics of cellular responses to perturbations that often depend on the logarithm of the dose. We estimate not only monotherapeutic dose–response curves but rather fit a complete dose–response surface to all of the data and use the resulting robust estimates of the monotherapeutic dose–response curves to construct a null reference response surface. Due to the probabilistic nature of Gaussian processes, we not only predict a (mean) synergy score for each dose combination but also a confidence interval for these scores. The model can be extended to incorporate more inputs, such as time or location, and is flexible enough to function as a building block in a pipeline that predicts cellular response to multiple perturbations.

## Methods

### Gaussian process dose–response model

**Fig. 18:**
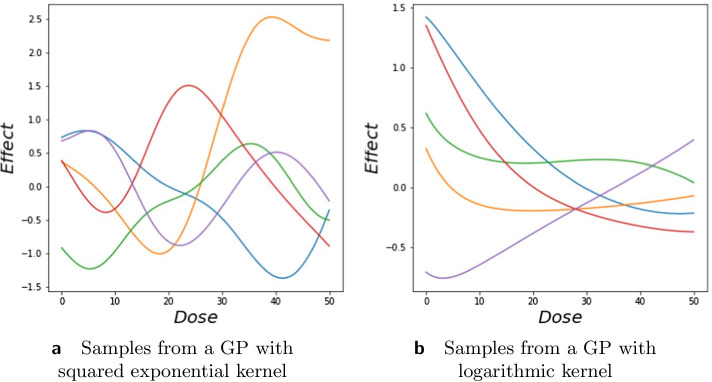
Samples from Gaussian process with logarithmic kernel. Different colors represent different samples. **a** Samples from the logarithmic squared exponential kernel with $l=10.0$ and $\sigma ^{2}_{f}=1.0$. **b** Samples from the squared exponential kernel with $l=10$ and $\sigma ^{2}_{f}=1.0$

In this section, we construct Gaussian process (GP) models for dose–response data. The models are based on GP regression with multi-dimensional input. For ease of understanding we start with the univariate model. Let *x* be a dose and *y* the response. We assume that the dose–response relationship follows

$$\begin{aligned} y = f(x) + \epsilon \, , \end{aligned}$$

where $\epsilon$ is a noise term and *f*(*x*) is represented by a GP: $f(x)\sim GP(0, k(x, x'))$ with $k(\cdot ,\cdot )$ a kernel function. A common choice of kernel for smooth functions is the squared exponential:

1 $$\begin{aligned} k(x,x') = \sigma _{f}^{2} \exp \left( -\frac{(x-x')^{2}}{2l^{2}}\right) , \end{aligned}$$

where $\sigma _{f}^{2}$ and *l*, correspondingly the variance and the length scale, are hyperparameters of the kernel. The variance determines how far from its mean the GP function deviates on average, and the length scale determines the smoothness of the function. The squared exponential kernel is stationary, and defines an infinitely differentiable function. Micchelli et al. [36] show that this kernel is universal in the sense that under some conditions, this kernel can learn any continuous structure given enough data [37]. The disadvantage of this kernel for dose–response modeling is that usually responses are affected by the logarithm of the dose, and a simple log transformation of dose is not desirable since we would need to take the logarithm of zero as dose zero is typically included in an experiment. We solve this problem by proposing the following kernel:

2 $$\begin{aligned} k_{\text {log}}(x,x') = \sigma _{f}^{2}\exp \left( -\frac{1}{2}\left( \log \left( 1+\frac{x}{l}\right) -\log \left( 1+\frac{x'}{l}\right) \right) ^{2}\right) , \end{aligned}$$

where *l* still has the interpretation of a length scale and $\sigma _{f}^{2}$ the amplitude of the GP function. Equation (2) defines a kernel for individual dose–response functions, i.e., for input $x \in {\mathbb {R}}$, *f*: ${\mathbb {R}}\rightarrow {\mathbb {R}}$. For doses $\{x,x'\} \ll l$, we have $\log (1 + x/l) \approx x/l$, and the kernel (2) is indistinguishable from the squared exponential kernel (1). Figure 18 shows samples from the squared-exponential and logarithmic kernels.

The generalization to bivariate input is straightforward. Let ${\varvec{x}}= (x_{1},x_{2}) \in {\mathbb {R}}^2$, $f: {\mathbb {R}}^{2}\rightarrow {\mathbb {R}}$, then $f({\varvec{x}}) = GP(0, k_{\text {log}}({\varvec{x}}, {\varvec{x}}'))$, where

3 $$\begin{aligned} k_{\text {log}}({\varvec{x}}, {\varvec{x}}')= & {} k_{\text {log}}(x_{1},x_{1}') \times k_{\text {log}}(x_{2}, x_{2}') \nonumber \\= & {} \sigma ^{2}_{f} \exp \left( -\frac{1}{2} \sum _{i=1}^2 \left( \log \left( 1+\frac{x_{i}}{l_{i}}\right) -\log \left( 1+\frac{x_{i}'}{l_{i}}\right) \right) ^{2}\right) . \end{aligned}$$

The kernel in Eq. (3) is constructed by multiplying two logarithmic kernels. This kernel structure intuitively means that $f(x_{1}, x_{2})$ is only expected to be similar to some other function value $f(x_{1}',x_{2}')$ if $x_{1}$ is close to $x_{1}'$ and $x_{2}$ is close to $x_{2}'$. Note that in Eq. (3) each dimension has its own length-scale parameter. In the dose–response framework the ratio of these length scales $l_{1}/l_{2}$ corresponds to the potency ratio, i.e., the ratio with which two drugs can substitute each other. The complete observational model with noise now reads

4 $$\begin{aligned} y = f(x_{1},x_{2}) + \epsilon , \end{aligned}$$

where $\epsilon \sim \mathcal{N}(0, \sigma ^{2})$ with hyperparameter $\sigma ^{2}$ the noise strength. In all, the bivariate GP model has four hyperparameters $\varvec{\theta }= \{\sigma ,\sigma _f,l_1,l_2\}$.

Let us denote by ${\varvec{y}}=(y_1,\dots ,y_n)$ the observations $y_{i}$ corresponding to the dose combinations ${\varvec{x}}_i=(x_{1i}, x_{2i})$, which are combined into matrix *X*. Let ${\varvec{x}}^{*}=(x_{1}^{*}, x_{2}^{*})$ be a test point with corresponding prediction $f^{*}$. Following Rasmussen et al. [18], we have

5 $$\begin{aligned} \left. \begin{bmatrix} {\varvec{y}}\\ f^{*} \end{bmatrix} \right| X, {\varvec{x}}^{*} \sim \mathcal{N}\Bigg (0, \begin{bmatrix} K + \sigma ^{2}{\mathbb {I}} &{} {\varvec{k}}\\ {\varvec{k}}^T &{} k^{*} \end{bmatrix}\Bigg ) \, , \end{aligned}$$

where *K* is the $n \times n$ kernel matrix with elements $K_{ij} = k_{\text {log}}({\varvec{x}}_i,{\varvec{x}}_j)$, ${\varvec{k}}$ an $n \times 1$ vector with elements $k_i = k_{\text {log}}({\varvec{x}}_i,{\varvec{x}}^{*})$, and $k^{*} = k_{\text {log}}({\varvec{x}}^{*},{\varvec{x}}^{*})$. This leads to the posterior distribution

6 $$\begin{aligned} f^{*} | {\varvec{x}}^{*},{\varvec{y}},X \sim \mathcal{N}({\hat{\mu }},{\hat{k}}) \text{ with } {\hat{\mu }} = {\varvec{k}}^T (K+\sigma ^{2}{\mathbb {I}})^{-1}{\varvec{y}} \text{ and } {\hat{k}} = k^{*} - {\varvec{k}}^T (K+\sigma ^{2}{\mathbb {I}})^{-1} {\varvec{k}}\, . \end{aligned}$$

Given hyperparameters and a data set with responses for various dose combinations, we can use Eq. (6) to compute the posterior mean response and credible intervals for any possible dose combination ${\varvec{x}}^{*}$.

We used a Bayesian approach to find the posterior distributions of the hyperparameters by sampling the hyperparameters using Hamiltonian Monte Carlo (HMC) methods [38]. A HMC sampling algorithm uses gradients of the target distribution, which allows for much more efficient exploration of the parameter space than MCMC algorithms relying on random walk proposals. We used the TensorFlow Probability library for the implementation of HMC. We ran the algorithm with 1000 burn-in steps and 100,000 samples. Target acceptance rate was set to 75%. Note that this acceptance rate is high for standard MCMC algorithms with random walk proposals such as Metropolis-Hastings, but common for MCMC algorithms that use Hamiltonian dynamics. We found that 5 leapfrog steps worked well in all of the applications considered in this paper. The proposal step size was tuned individually and chosen from the set $\{0.01; 0.02; 0.03; 0.05; 0.08; 0.1; 0.2; 0.3; 0.6; 1.00\}$ which is an equally spaced sequence on the logarithmic scale. In each case the largest step size that achieved the target acceptance rate was chosen. We used weakly informative priors for the hyperparameters of the kernel. We used a Gamma$(\alpha , \beta )$ distribution for the hyperparameters of the kernel, length scale, and variance. For the length scale we chose $\alpha$ and $\beta$ such that $\alpha / \beta$ is equal to the maximal dose of a drug scaled by a constant *c* and $\alpha /\beta ^{2}$ is $0.1\times$half of the maximal dose of the drug scaled by a constant *c*. Constant *c* reflects whether the effect changes in the observed data points. One strategy to set *c* is to set it to the ratio between maximal and minimal effect reached on the monotherapeutic data. For the variance we choose $\alpha$ and $\beta$ such that $\alpha /\beta$ is equal to half of the maximum effect reached and $\alpha /\beta ^{2}$ is equal to $0.1\times$half of the maximum effect reached. A Gamma(0.14, 1.14) prior was used for the noise variance in all applications except the Greco data. For the Greco data we used a Gamma(2, 1) prior. This difference is due to the different scale of the data. Examples of the samples obtained with HMC can be found in the Additional file 1: Fig. S3. As one can see, the chains are well mixed and converged. The uncertainty about the parameter values is based on the 95% highest posterior density intervals obtained from the HMC samples.

### Null reference model from the Hand principle

We are interested in whether two drugs are *synergetic* or *antagonistic*, i.e. whether the mixture has stronger/weaker effect compared to what we would have expected if the drugs had no interaction. Null reference models are designed such that there is no interaction between drugs. There are three desirable properties that null reference models should satisfy [8]:

- *Sham combination principle* a drug does not interact with itself. Hence the combination of a drug with itself leads to neither synergy nor antagonism;
- *Commutativity* swap of drugs should not change the results;
- *Associative property* combining a combination drugs should be the same as directly combining the drugs at their corresponding ratios.

**Fig. 19:**
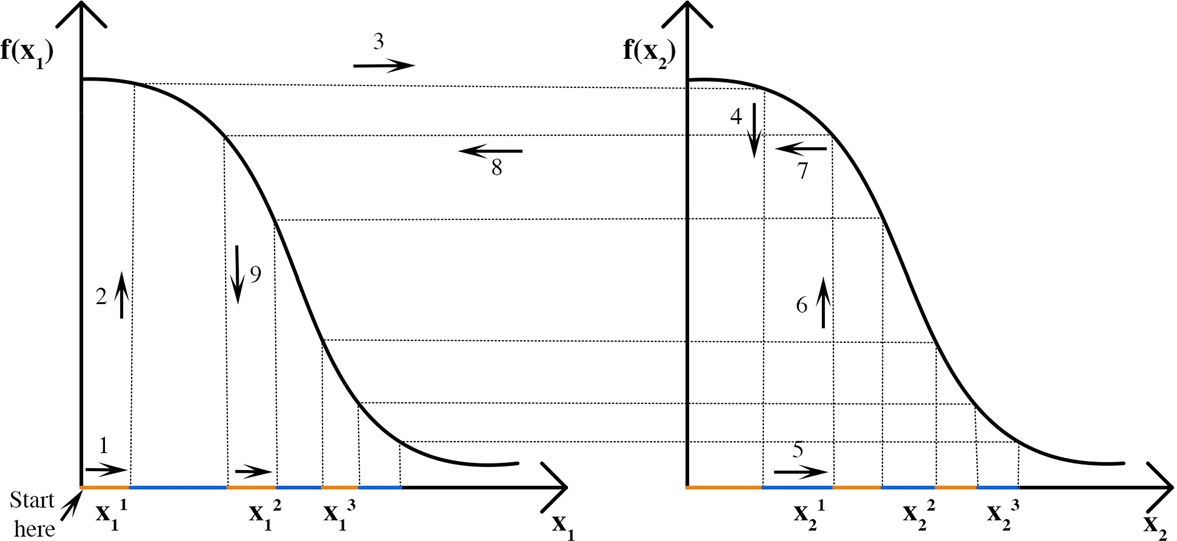
Illustration of the Hand model. In this case doses $x_{1}$ and $x_{2}$ are split into $N_1=N_2=3$ parts and the partitions are applied sequentially. The application of the first two partitions $x_{1}^{1}$ and $x_{2}^{1}$ is guided by the arrows 1-9, after that the process continues in the same way until all partitions of both drugs are applied. The partitions of the drug 1 are illustrated in orange and the partitions of the drug 2 are illustrated in blue

Sinzger et al. 2019 [8] compare various popular null reference models and show that the Hand model is biochemically the most plausible as it is the only model satisfying all three desirable properties. The Hand model construction is close to that of Loewe, which is detailed in Lederer et al. [39]. The Hand model can be viewed as an infinitesimal version of the Loewe model, thereby solving the commutativity issues that plague models based on Loewe additivity. In Algorithm 1 we present the implementation of the Hand-GP model and in Fig. 19 we provide an illustration of the Hand construction. In the current implementation, Algorithm 1 requires $f(\cdot )$ to be invertible. Note that $f^{-1}(\cdot )$ has to be approximated numerically since it is impossible to find the inverse of a Gaussian process analytically. However, if we use a large enough number of test points for predicting a Gaussian process, we can get a very good numerical approximation. For Algorithm 1 we need to choose $N_{1}$, the number of partitions of dose $x_{1}$, and $N_{2}$, the number of partitions of dose $x_{2}$. We choose $N_{1}$ and $N_{2}$ depending on the number of test points for the Gaussian process model and where the applied doses $x_{1}$ and $x_{2}$ are located among these test points. By test points we mean here the points in which we make predictions for the Gaussian process regression, denoted as ${\varvec{x}}^{*}$. For choosing $N_{1}$ we find the closest point among the test points to $x_{1}$ and choose $N_{1}$ as the number of test points between 0 dose and closest test point to $x_{1}$. $N_{2}$ is chosen in the same way considering dose $x_{2}$.

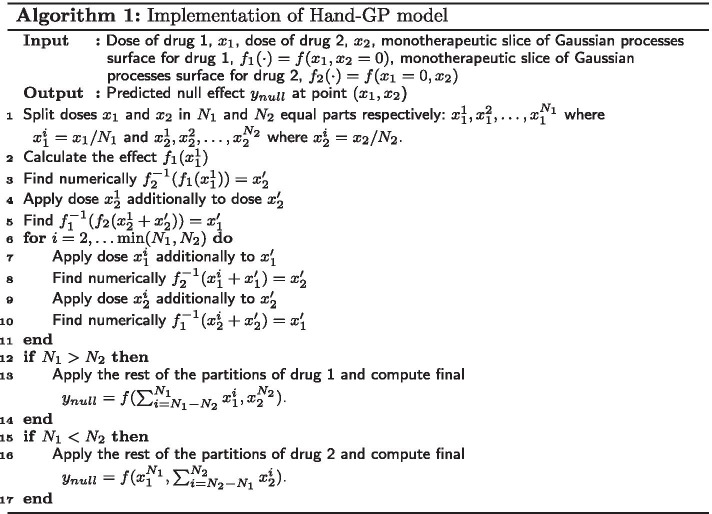



### MuSyC model

The MuSyC model was fitted using the Python library synergy [21]. We constrained certain parameters when optimizing the MuSyC model to make sure that the confidence intervals for the model parameters are reasonably estimated. We imposed the following constraints: $E_{max}$ is within limits [0,100] or [0,1] depending on the application, we limited the parameters $E_{1}, E_{2}, E_{3}$ to be in [0, 100] or [0, 1]. Further, when estimating the null MuSyC model we imposed the constraints: $\alpha _{12}=\alpha _{21}=\gamma _{12}=\gamma _{21}=1$ and $\beta =0$. Confidence intervals for the surface of the MuSyC model were obtained with the parametric bootstrap [22]. We did not provide confidence intervals for the surface of the null MuSyC model and the summary measures which are dependent on it since the synergy library does not provide confidence intervals on the parameters of the null model.

## Supplementary Information

**Additional file 1.** Non-parametric synergy modelingof chemical compounds with Gaussian processes: Supplementary figures.

## Data Availability

Five benchmark data sets and the source code are available at https://github.com/YuliyaShapovalova/HandGP. Archived version: 10.5281/zenodo.5148284. The anti-cancer drug combination screen is available from the supplemental materials of O’Neil et al. [28], (10.1158/1535-7163.MCT-15-0843). Mott et al. anti-malarial screen was downloaded from the repository of the recent paper by Wooten et al. [17] https://bitbucket.org/meyerct1/musyc_theory/ and the raw data are available from publicly available sources with (10.1038/srep13891).

## Abbreviations

GP: Gaussian processes
MuSyC: Multi-dimensional synergy of combinations
HSA: Highest Single Agent
MSE: Mean squared error
HMC: Hamiltonian Monte Carlo
MCMC: Markov Chain Monte Carlo
