# Supplementary material for: Non-parametric synergy modeling of chemical compounds with Gaussian processes
Source: BMC Bioinformatics. 2022 Jan 6;23:14. doi: 10.1186/s12859-021-04508-7 (PMC8734200; doi:10.1186/s12859-021-04508-7)
Supplement: Supplementary file 1 — Additional file 1. Non-parametric synergy modelingof chemical compounds with Gaussian processes: Supplementary figures. [file 12859_2021_4508_MOESM1_ESM.pdf]

NON-PARAMETRIC SYNERGY MODELING OF CHEMICAL COMPOUNDS WITH GAUSSIAN PROCESSES: SUPPLEMENTARY FIGURES

Comparison of squared exponential and logarithmic kernel

Table S1. Comparison of Gaussian processes fits on different data sets with squared exponential and logarithmic kernels. Lowest MSE for each drug is indicated in **bold**

Data Set	MSE sq. exp. drug A	MSE sq. exp. drug B	MSE log. drug A	MSE log. drug B	Scale of data	Scale of doses
Greco	91.52	72.15	**72.01**	**63.35**	[0,100.0]	Logarithmic
Loewe synergy	**1.05** $\times 10^{-6}$	**1.05** $\times 10^{-6}$	1.17 $\times 10^{-4}$	1.17 $\times 10^{-4}$	[0,100.0]	Linear
Loewe antagonism	**4.9** $\times 10^{-6}$	**4.9** $\times 10^{-6}$	1.21 $\times 10^{-4}$	1.21 $\times 10^{-4}$	[0,100.0]	Linear
Chou Talalay 1	3.3 $\times 10^{-4}$	**6.8** $\times 10^{-4}$	**2.9** $\times 10^{-4}$	6.3 $\times 10^{-4}$	[0,1.0]	Logarithmic
Chou Talalay 2	82.61	64.07	**55.28**	**45.26**	[0,1.0]	Logarithmic

Simulated data Loewe synergy and Loewe antagonism

We use the approach of Wicha et al.[2] to generate bidirectional synergistic and antagonistic data sets. We use code provided with the paper to generate the data [2] using the following parameters: 1) synergy $Emax_1 = 1, EC50_1 = 50, H_1 = 4, Emax_2 = 1, EC50_2 = 50, H_2 = 4, Int_{12} = -.9, Int_{21} = -.9, EC50Int_{12} = 25, EC50Int_{21} = 25$; 2) antagonism $Emax_1 = 1, EC50_1 = 50, H_1 = 4, Emax_2 = 1, EC50_2 = 50, H_2 = 4, Int_{12} = 1, Int_{21} = 1, EC50Int_{12} = 25, EC50Int_{21} = 25$. No noise was added to these data.

ASSESSING THE MODELS ON DRUG COMBINATION SCREENS: EXAMPLES FROM THE O'NEIL ET AL. ANTI-CANCER SCREEN [3]

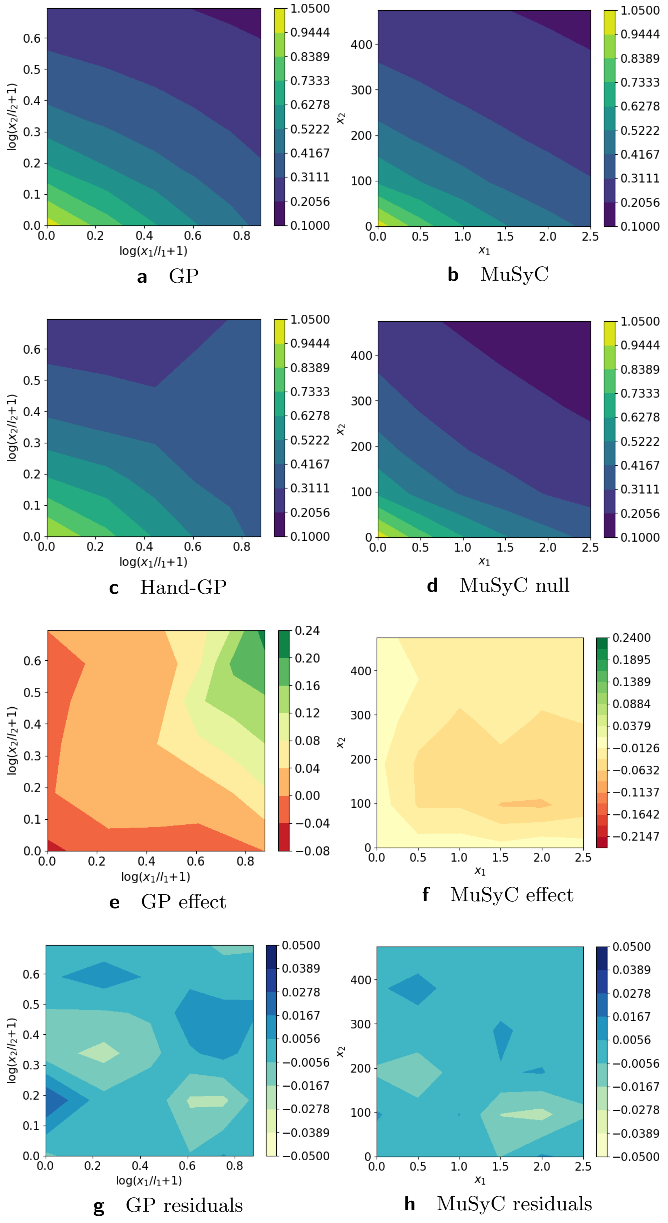

Fig. S1. Analysis of the mutually exclusive inhibitors data set[1] with the Hand-GP (left column) and MuSyC (right column) models. Top row shows the fitted response surfaces, for Hand-GP this is a fit to the non-parametric GP model for MuSyC a fit to the parametric MuSyC model. The second row shows the null reference models. For Hand-GP this is the Hand construction derived from the fitted monotherapeutic responses from the top row. For the MuSyC model this is a fit to a constrained MuSyC model. The third row shows the synergistic effect surfaces as difference between the first and second row. The bottom row shows the residuals, the difference between the data and the fits from the top row.

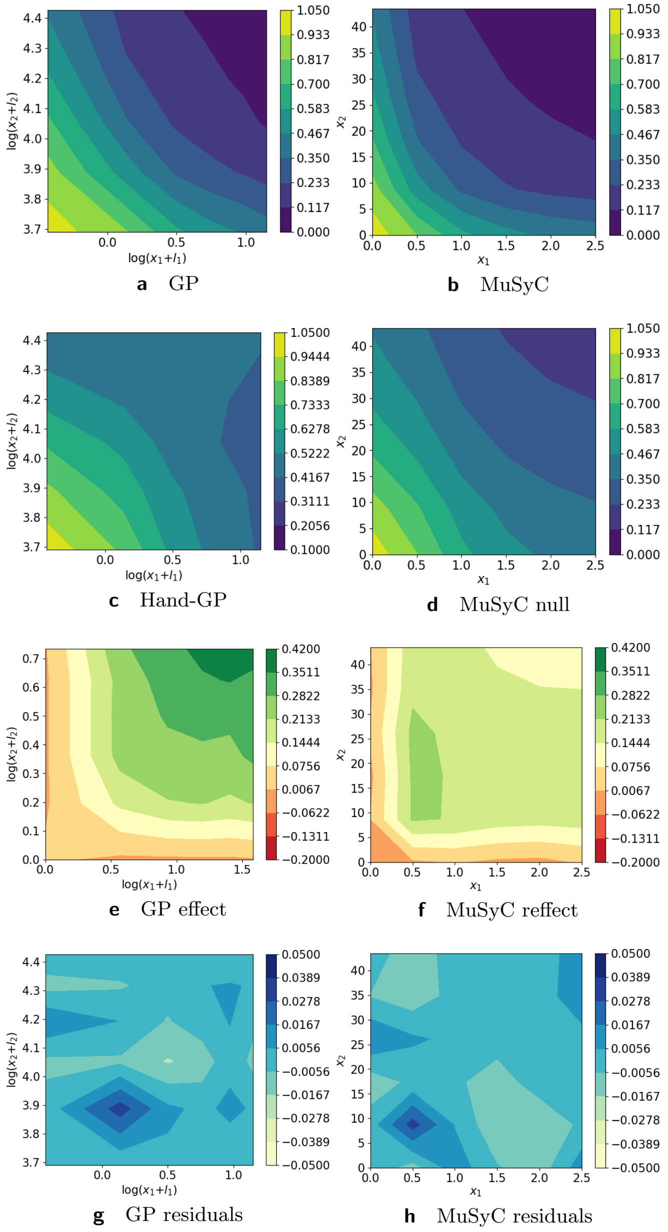

Fig. S2. Analysis of the mutually non-exclusive inhibitors data set[1] with Hand-GP (left column) and MuSyC (right column) models. Top row shows the fitted response surfaces, for Hand-GP this is a fit to the non-parametric GP model for MuSyC a fit to the parametric MuSyC model. The second row shows the null reference models. For Hand-GP this is the Hand construction derived from the fitted monotherapeutic responses from the top row. For the MuSyC model this is a fit to a constrained MuSyC model. The third row shows the synergistic effect surfaces as difference between the first and second row. The bottom row shows the residuals, the difference between the data and the fits from the top row.

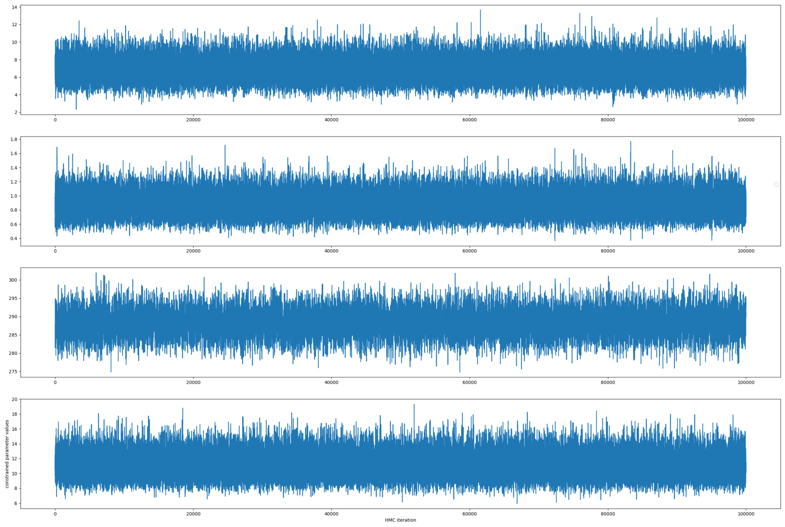

a Trace plots of the MCMC samples for Greco data

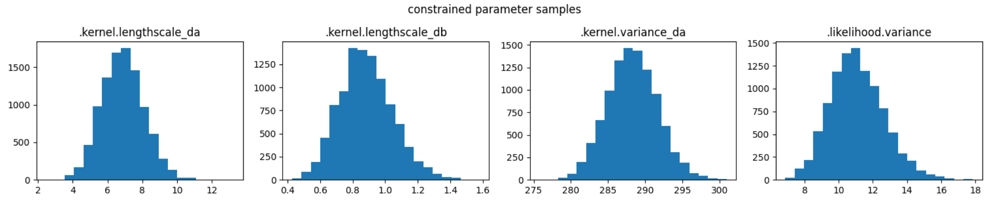

b Histograms of the MCMC samples for Greco data

Fig. S3. HMC results for the Greco data set. The samples are obtained using Hamiltonian Monte Carlo with leap frog step 5, step size 0.7, target acceptance rate 0.75 and adaptation rate 0.1. Since the number of the hyperparameters is small, it is not hard to get good performance of HMC in terms of convergence and mixing.

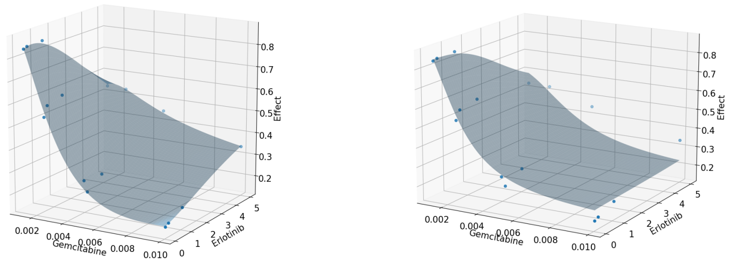

a GP effect **b** MuSyC effect

Fig. S4. Estimated surfaces for the combination of Gemcitabine and Erlotinib for isolate HB30 (O'Neil et al. anti-cancer screen). Left column shows the estimated surface of the Hand-GP model and right column the estimated surface of the MuSyC model.

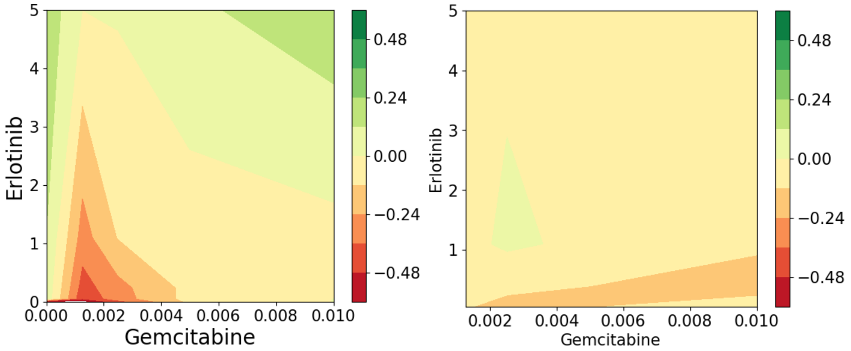

a GP effect **b** MuSyC effect

Fig. S5. Predicted effect for the combination of Gemcitabine and Erlotinib for isolate HB30 (O'Neil et al. anti-cancer screen). On the left prediction by the Hand-GP model, on the right prediction by the MuSyC model.

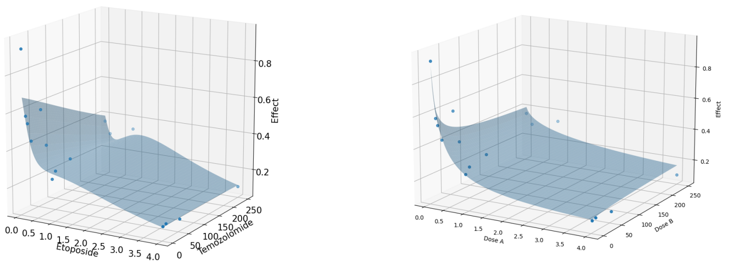

a GP effect **b** MuSyC effect

Fig. S6. Estimated surfaces for the combination of Etoposide and Temozolomide for isolate HB30 (O'Neil et al. anti-cancer screen). Left column shows the estimated surface of the Hand-GP model and right column the estimated surface of the MuSyC model.

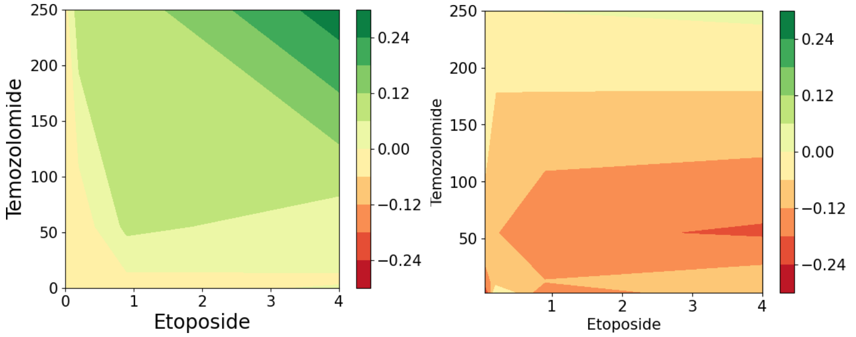

a GP effect **b** MuSyC effect

Fig. S7. Predicted effect for the combination of Etoposide and Temozolomide for isolate HB30 (O'Neil et al. anti-cancer screen). On the left prediction by the Hand-GP model, on the right prediction by the MuSyC model.

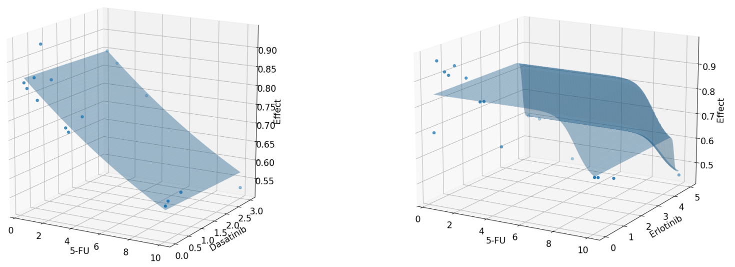

a GP effect **b** MuSyC effect

Fig. S8. Estimated surfaces for the combination of 5-FU and Dasatinib for isolate HB30 (O'Neil et al. anti-cancer screen). Left column shows the estimated surface of the Hand-GP model and right column the estimated surface of the MuSyC model.

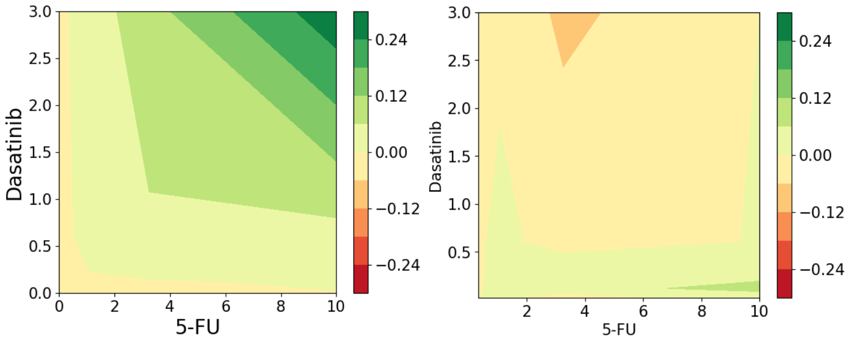

a GP effect **b** MuSyC effect

Fig. S9. Predicted effect for the combination of 5-FU and Dasatinib for isolate HB30 (O'Neil et al. anti-cancer screen). On the left prediction by the Hand-GP model, on the right prediction by the MuSyC model.

REFERENCES

1. T. Yonetani and H. Theorell, “Studies on liver alcohol dehydrogenase complexes: III. multiple inhibition kinetics in the presence of two competitive inhibitors,” Arch. biochemistry biophysics **106**, 243–251 (1964).
2. S. G. Wicha, C. Chen, O. Clewe, and U. S. Simonsson, “A general pharmacodynamic interaction model identifies perpetrators and victims in drug interactions,” Nat. communications **8**, 2129 (2017).
3. J. O’Neil, Y. Benita, I. Feldman, M. Chenard, B. Roberts, Y. Liu, J. Li, A. Kral, S. Lejnine, A. Loboda *et al.*, “An unbiased oncology compound screen to identify novel combination strategies,” Mol. cancer therapeutics **15**, 1155–1162 (2016).
